# First mitogenome phylogeny of the sun bear *Helarctos malayanus* reveals a deep split between Indochinese and Sundaic lineages

**DOI:** 10.1002/ece3.9969

**Published:** 2023-04-18

**Authors:** Miriam N. Kunde, Axel Barlow, Achim M. Klittich, Aliya Yakupova, Riddhi P. Patel, Jörns Fickel, Daniel W. Förster

**Affiliations:** ^1^ Leibniz Institute for Zoo and Wildlife Research Alfred‐Kowalke‐Str. 17 10315 Berlin Germany; ^2^ School of Environment Griffith University Nathan Campus, 170 Kessels Road, Nathan Brisbane Queensland 4111 Australia; ^3^ School of Natural Sciences Bangor University Bangor Gwynedd LL57 2DG UK; ^4^ Institute for Biochemistry and Biology University of Potsdam Karl‐Liebknecht‐Str. 24–25 14476 Potsdam Germany; ^5^ Computer Technologies Laboratory ITMO University 197101 Saint Petersburg Russia

**Keywords:** biogeography, *Helarctos malayanus*, intraspecific variation, mitogenome, sun bear

## Abstract

The sun bear *Helarctos malayanus* is one of the most endangered ursids, and to date classification of sun bear populations has been based almost exclusively on geographic distribution and morphology. The very few molecular studies focussing on this species were limited in geographic scope. Using archival and non‐invasively collected sample material, we have added a substantial number of complete or near‐complete mitochondrial genome sequences from sun bears of several range countries of the species' distribution. We here report 32 new mitogenome sequences representing sun bears from Cambodia, Thailand, Peninsular Malaysia, Sumatra, and Borneo. Reconstruction of phylogenetic relationships revealed two matrilines that diverged ~295 thousand years ago: one restricted to portions of mainland Indochina (China, Cambodia, Thailand; “Mainland clade”), and one comprising bears from Borneo, Sumatra, Peninsular Malaysia but also Thailand (“Sunda clade”). Generally recent coalescence times in the mitochondrial phylogeny suggest that recent or historical demographic processes have resulted in a loss of mtDNA variation. Additionally, analysis of our data in conjunction with shorter mtDNA sequences revealed that the Bornean sun bear, classified as a distinct subspecies (*H. m. euryspilus*), does not harbor a distinctive matriline. Further molecular studies of *H. malayanus* are needed, which should ideally include data from nuclear loci.

## Graphical abstract

Using archival and non‐invasively collected sample material, we recovered mitochondrial genome sequences from several range countries of the sun bear *Helarctos malayanus*. Reconstruction of phylogenetic relationships revealed two matrilines: one restricted to portions of mainland Indochina (China, Cambodia, Thailand; “Mainland clade”), and one comprising bears from Borneo, Sumatra, Peninsular Malaysia but also Thailand (“Sunda clade”). The Bornean sun bear, classified as a distinct subspecies (H. m. euryspilus), does not harbor a distinctive mtDNA lineage.

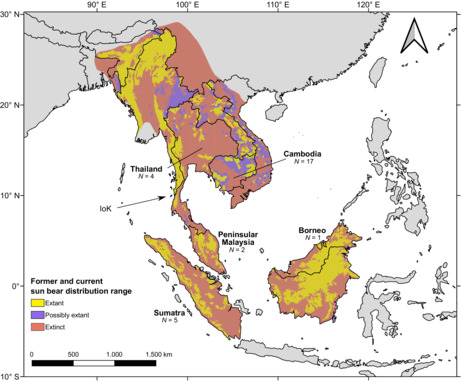

## 1 INTRODUCTION

Archival and non‐invasively collected sample material is increasingly utilized in molecular studies of wildlife species, particularly if these are rare, elusive, protected, or inhabit areas that are difficult to access (e.g., Cho et al., 2022; Hessels et al., 2021; Mengüllüoğlu et al., 2021; Paijmans et al., 2020; Sacks et al., 2021; von Thaden et al., 2021). DNA extracted from such material is usually highly degraded, rendering downstream analyses difficult (e.g., Pääbo, 1989). However, targeted capture coupled with high throughput sequencing enables retrieval of genetic information from such degraded sample material, with mitochondrial DNA (mtDNA) often the marker of choice, because the high copy number of mitochondria per cell benefits sequence recovery (e.g., Jones & Good, 2016; Paijmans et al., 2013). MtDNA sequences enriched by targeted capture can then be used to resolve population genetic structure and taxonomic relationships within species, and can provide insights into the historical processes that have shaped contemporary populations (Avise, 2000).

With regard to threatened species, conservation management greatly benefits from such knowledge (Martins, Schmidt, et al., 2017; Wilting et al., 2011, 2015), as observational data or phenotypic characters may not accurately reflect how genetic variation is partitioned within species (Martins, Fickel, et al., 2017; Patel et al., 2017; Wilting et al., 2016), or which populations have evolved independently for long periods of time (‘evolutionary significant units’). Such information is still lacking for many wildlife species, including those inhabiting ecosystems in global biodiversity hotspots, such as Southeast (SE) Asia, that are increasingly adversely affected by anthropogenic land‐use and land‐cover change (Nguyen et al., 2021; Tilker et al., 2020).

**FIGURE 1:**
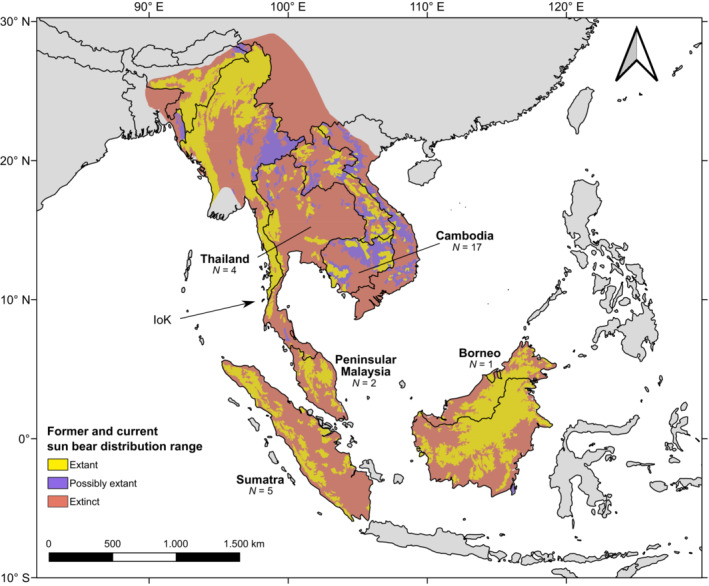
Former and current *H. malayanus* distribution range (following Scotson et al., 2017). The number of samples for which we were able to retrieve mitochondrial genomes is indicated, as is the Isthmus of Kra (“IoK”).

Such a knowledge gap also exists for the Malayan sun bear *Helarctos malayanus*, which is currently listed as Vulnerable on the IUCN Red List of threatened species (Scotson et al., 2017) and is in Appendix 1 of CITES (CITES, 2022). Sun bears perform significant ecological functions throughout their range, including soil turnover, seed dispersal, and the maintenance of trophic relationships (e.g., Augeri, 2005; Mcconkey & Galetti, 1999). This monotypic species of small bears was once widely distributed across SE Asia (Figure 1), occurring in eastern India, Bangladesh, Myanmar, Thailand, Laos, Cambodia, Vietnam, Malaysia, Indonesia, and southern China (Fitzgerald & Krausman, 2002). Over the past decades, the species' distribution area has dramatically shrunk, and it is estimated that sun bears now only occur in 32%–40% of their former range (Crudge et al., 2019), with a concomitant decline in population size (e.g., Crudge et al., 2016; Jenks et al., 2011; Scotson et al., 2017). It is the sole ursid that resides in the Sundaic region, which has experienced significant forest loss in recent years, and population reductions in this region are anticipated to be the most severe (Scotson et al., 2017).

In order to describe and properly manage the remaining genetic diversity in sun bears molecular data are needed. For example, a phylogeographic approach can reveal management and evolutionary units (Avise, 1992; Moritz, 1994), help to identify conservation priorities (Goossens et al., 2013), and pinpoint suitable reintroduction locations (Apollonio et al., 2014).

Existing studies of *H. malayanus* using molecular markers (mtDNA sequences and microsatellites) were limited in their geographic scope (Kunde et al., 2020; Lai, Chew, et al., 2021; Onuma et al., 2006), and we thus lack knowledge about the distribution of intraspecific genetic diversity across the species' range. To address this shortcoming, we sequenced mitochondrial genomes from samples across a large portion of the sun bear distribution, utilizing both archival material from natural history museums as well as non‐invasively collected material (mucosal cells from saliva). Using these complete mitochondrial genomes (mitogenomes), we elucidated the geographic distribution of maternal lineages (matrilines) and provide insights into the species' history.

## 2 MATERIALS AND METHODS

### 2.1 Samples

Dried tissue remains from the skull or tissue from the nasal cavity of 29 archival sun bear specimens were collected from several natural history museums (Table S1), providing a broad geographic distribution of samples.

We also collected saliva samples from 21 sun bears, which originated from 12 provinces in Cambodia and which were housed at the ‘Free the Bears’ sanctuary in Phnom Tamao Zoo and Wildlife Rescue Centre. We included up to two samples per province in order to obtain a balanced geographic coverage of Cambodia.

### 2.2 Archival and non‐invasively collected DNA

DNA from archival samples was extracted following Wilting et al. (2016), in a laboratory designed for and limited to the use of archival material. DNA extractions were conducted in batches consisting of three samples and one negative control and verified to be of sun bear origin by amplification of a 146 bp long portion of the mitochondrial cytochrome *b* gene using species specific primers (Table S2).

DNA extractions from non‐invasively collected samples (salivary mucosal cells) were carried out using the GEN‐IAL First DNA *All*‐tissue DNA extraction Kit (GEN‐IAL GmbH) following manufacturer's instructions. This work was carried out in a separate facility, dedicated to molecular work with non‐invasively collected material.

### 2.3 Library preparation and hybridization capture

Illumina sequencing libraries were constructed following Fortes and Paijmans (2015), using double‐indexing with 8‐bp indexes. Prior to indexing we conducted qPCRs to determine the optimal PCR cycle number for each sample.

To reduce effects by target DNA degradation and external DNA contaminations, we carried out hybridisation capture for both sample types (archival, non‐invasively collected).

Baits for hybridization capture were generated using long range (LR) PCR products that spanned three large overlapping regions of the mitogenome (using a fresh *Helarctos malayanus* sample as template). The LR‐PCR primers were designed using the genbank reference sequence NC_009968: HMA1‐F (5′‐ACGACCTCGATGTTGGATCAGG‐3′) and HMA1‐R (5′‐AGGGCTACAGCGAACTCGAGA‐3′), yielding a 6152 bp long product, HMA2‐F (5′‐GCCACACTCATTCACACCTACCA‐3′) and HMA2‐R (5′‐AGTCCTTTCTGGTTGGAGACTGTG‐3′), yielding a 5299 bp long product, and HMA3‐F (5′‐ACCAACGCCTGAGCCCTACT‐3′) and HMA3‐R (5′‐GCGCTTTAGTGAGGGAGGCC‐3′), yielding a 6416 bp long product. Amplifications were carried out in 50 μL reaction volumes: 18 μL dH_2_O, 25 μL Bioline Myfi mix (Bioline GmbH), 2 μL of forward and reverse primers (10 μM), 3 μL of template DNA (100 ng/μL). PCR conditions were as follows: initial denaturation at 94°C for 3 min, followed by 40 cycles of denaturation at 94°C for 30 s; annealing at 60°C for 30 s; extension at 68°C for 7 min with the final extension at 68°C for 10 min.

LR‐PCR products were sheared (target size 300 bp), pooled equimolarly and then converted into biotinylated baits following Maricic et al. (2010). Targeted capture was carried out using hybridization temperatures appropriate for sample type (Paijmans et al., 2016). Paired‐end sequencing was carried out on the Illumina MiSeq platform (Illumina) using v3 150‐cycle kits.

### 2.4 Bioinformatic workflow

We de‐multiplexed paired‐end reads using bcl2fastq v2.17.1.14 (Illumina, Inc.) and removed adapter sequences using cutadapt v1.3 (Martin, 2011). Using trimmomatic (Bolger et al., 2014), we applied a sliding window approach for quality trimming with the phred quality threshold set at *Q* = 20. Next, we merged the adapter‐clipped and quality‐trimmed sequences using flash v1.2.8 (Magoc & Salzberg, 2011). Merged sequences were then mapped to the reference sun bear mitogenome sequence (GenBank accession no. NC_009968) with the BWA aln algorithm v0.7.10 (Li & Durbin, 2009). Mapped sequences were then de‐duplicated using markduplicates from picard‐tools v1.106 (https://github.com/broadinstitute/picard), followed by variant calling using samtools v1.1 (Li et al., 2009) and bcftools v1.2 (http://github.com/samtools/bcftools). Positions along the mitogenome were N‐masked if their sequence depth was below 5×, or when they did not conform to an 80% majority rule for base calling.

### 2.5 Summary statistics

The final data set of complete or near‐complete sun bear mitogenomes consisted of 15 archival and 17 non‐invasively collected samples, analyzed together with three published sequences (GenBank: MN807949, FM177765 and EF196664; see Table 1 for details).

**TABLE 1:** Sample details.

Sample ID	Region	Sample type	Raw reads	Clipped, trimmed and merged sequences	Deduplicated sequences mapped to mitogenome	% mitogenome covered ≥5×
HMA_1_MA	Peninsular Malaysia	Archival	962,556	395,594	7632	97.95
HMA_2_MA	Peninsular Malaysia	Archival	4,279,758	1,958,381	325,844	99.92
HMA_4_TH	Thailand	Archival	782,922	357,378	7550	95.96
HMA_5_SU	Sumatra	Archival	655,610	300,916	74,221	98.26
HMA_13	*Unknown*	Archival	703,722	319,130	43,562	96.89
HMA_15_TH	Thailand	Archival	1,999,180	881,484	172,478	98.64
HMA_16_SU	Sumatra	Archival	1,431,992	643,106	56,190	98.49
HMA_17_SU	Sumatra	Archival	1,214,110	548,477	20,344	97.89
HMA_19_BO	Borneo	Archival	998,120	407,017	28,629	98.87
HMA_24_TH	Thailand	Archival	1,105,638	499,533	7984	97.72
HMA_26_TH	Thailand	Archival	1,119,718	521,872	149,064	98.71
HMA_21_CAMB	Cambodia	Saliva	632,824	285,401	72,500	98.96
HMA_27_CAMB	Cambodia	Saliva	842,154	349,635	34,873	99.03
HMA_28_CAMB	Cambodia	Saliva	853,854	366,541	10,990	98.77
HMA_31_CAMB	Cambodia	Saliva	707,374	273,830	8936	98.74
HMA_35_CAMB	Cambodia	Saliva	1,311,174	578,748	16,942	98.71
HMA_37_CAMB	Cambodia	Saliva	1,230,298	547,022	7365	98.12
HMA_42_CAMB	Cambodia	Saliva	860,156	362,656	12,187	98.81
HMA_46_CAMB	Cambodia	Saliva	1,816,556	766,221	36,603	98.89
HMA_52_CAMB	Cambodia	Saliva	1,623,318	585,438	10,331	98.71
HMA_57_CAMB	Cambodia	Saliva	992,996	441,147	8488	98.73
HMA_61_CAMB	Cambodia	Saliva	1,619,226	608,738	46,909	99.02
HMA_65_CAMB	Cambodia	Saliva	2,232,478	822,778	28,069	99.09
HMA_69_CAMB	Cambodia	Saliva	723,998	321,510	23,069	98.87
HMA_79_CAMB	Cambodia	Saliva	951,314	419,079	58,623	99.03
HMA_86_CAMB	Cambodia	Saliva	961,508	421,987	7186	95.14
HMA_87_CAMB	Cambodia	Saliva	954,048	375,369	17,300	98.99
HMA_95_CAMB	Cambodia	Saliva	1,073,632	370,083	8575	98.80
HMA_34002_SU	Sumatra	Archival	2,380,950	1,083,797	139,932	98.84
HMA_34004_SU	Sumatra	Archival	5,117,344	1,934,439	58,458	99.32
HMA_15638	*Unknown*	Archival	2,897,942	936,442	25,587	98.27
HMA_A5351	*Unknown*	Archival	4,472,384	1,667,180	19,935	98.49
MN807949	Peninsular Malaysia	*Genbank*	n.a.	n.a.	n.a.	n.a.
FM177765	*Unknown*	*Genbank*	n.a.	n.a.	n.a.	n.a.
EF196664	China	*Genbank*	n.a.	n.a.	n.a.	n.a.

*Note*: Further sample details are provided in Table S1.

Mitogenome sequences were aligned and manually curated in Geneious v. 8.1.9 (Kearse et al., 2012). Summary statistics for the complete dataset (*N* = 35), as well as subdivisions based on phylogenetic analyses (below) were generated using DnaSP v.6.12.03 (Rozas et al., 2017). For these estimates we excluded sites with missing data, ambiguous data, or gaps.

### 2.6 Phylogenetic analyses

To generate a time‐calibrated mitochondrial phylogeny of sun bears, we first estimated the root age in a species level analysis calibrated based on the fossil ages of other representatives of the Ursidae. We then applied this root age estimate to population level analyses of sun bear mitochondrial sequences. This two step strategy was chosen to best accommodate the assumptions of the tree priors used in these respective analyses.

For the species level analysis, we aligned two divergent sun bear mitogenomes (HMA_26_TH, and HMA_35_CAMB) and one mitogenome from each of the following nine species: *Ursus maritimus* (Polar bear; acc. no. AF303111), *Ursus arctos* (Brown bear; acc. no. HQ685956), *Ursus thibetanus* (Asian black bear; acc. no. EF196661), *Ursus americanus* (American black bear; acc. no. AF303109), *Melursus ursinus* (Sloth bear; acc. no. FM177763), *Tremarctos ornatus* (Spectacled bear; acc. no. EF196665), *Ailuropoda melanoleuca* (Giant Panda; acc. no. EF196663), *Arctodus simus* (extinct Short‐faced bear; acc. no. FM177762) and *Ursus ingressus* (extinct Cave bear; acc. no. KX641331). We then used PartitionFinder v2.1.1 (Lanfear et al., 2016) to identify an optimal set of partitions and substitution models among all combinations of tRNAs, rRNAs and the three codon positions of the protein coding genes, using the greedy search algorithm and considering all substitution models available in BEAST, using the Bayesian Information Criterion. The Bayesian phylogenetic analysis package BEAST v.1.8.2 (Drummond et al., 2012) was then used to estimate species phylogeny and divergence times (Figure S1). A birth‐death tree model was used, with a lognormal relaxed clock model, with a uniform prior on the mean substitution rate of 0 to 20% per million years (My). Time‐calibration was achieved using informative uniform priors based on fossil and other evidence, following the approach of a previous study of the Ursidae (Kumar et al., 2017). Monophyly was enforced for all calibrated nodes. These were: (1) a prior based on the divergence of the Tremarctinae and Ursinae clades between 7 and 14 My, based on a fossil of Tremarctine bear *Plionarctos* (Tedford, 2001); (2) a prior for the basal divergence of the Ursidae with an upper limit of 12 My, based on a fossil of the Ailuropodinae (Abella et al., 2012) and a lower limit of 20 My, determined by a molecular dating study (Wu et al., 2015); (3) a prior on divergence of the Ursinae between 4.3 and 6 My based on the age of *Ursus minimus* (Gustafson, 1978); and (4) a prior on the divergence of *U. arctos* and *U. maritimus* between 0.48 and 1.1 My based on previous studies (Cahill et al., 2015; Hailer et al., 2012; Li et al., 2011). All other priors were left at default values. Initial runs showed a lack of convergence for some substitution model parameters, and so we substituted them with simpler models (Table S3). The final BEAST run involved 20 million generations, sampling the MCMC chain every 1000 generations, in order to achieve adequate burn in and posterior sampling of all parameters (ESS >200), assessed using the program Tracer 1.7.

The divergence estimate recovered for the two sun bear mitogenomes (mean 0.3054 My, standard deviation 0.0371 My) was then applied as normal prior on the root height for the intraspecific analyses, and were run with a Bayesian Skyline coalescent population model with eight groups. Data partitions and substitutions were estimated using PartitionFinder as described previously. As we did not expect variation in mitochondrial substitution rate within sun bears, we used a strict clock model with a uniform prior on the per‐lineage substitution rate of 0 to 20% per My. Details of the MCMC runs were as described above. The maximum clade credibility tree was then selected from the posterior sample, and node heights scaled to the median posterior age, using TreeAnnotator 1.8.2.

Maximum‐likelihood phylogenetic analyses for both mitogenome datasets were performed using RAxML 8.2.12 (Stamatakis, 2014). Clade support was assessed using 100 rapid bootstrap replicates assuming the GTR + CAT substitution model, with a subsequent thorough maximum likelihood search for the best tree assuming the GTR + G substitution model. For the species level analysis, the tree was rooted using the Giant Panda (acc. no. EF196663) as an outgroup (Figure S2). For the intraspecific analysis, the Asian black bear (acc. no. EF196661) was used as an outgroup (Figure S3).

We also generated a dataset combining portions of our mitochondrial sequences with a set of recently published ~1800 bp long mtDNA sequences from sun bears from Thailand, Peninsular Malaysia and Borneo (GenBank acc. no. MW316324–MW316405; Lai, Chew, et al., 2021). This combined dataset of shorter sequences consisted of 117 mtDNA sequences: 32 from this study, 82 from Lai, Chew, et al. (2021), one from Lai, Ratnayeke, et al. (2021), one from Yu et al. (2007), and one from Krause et al. (2008; Table S4). The alignment had a length of 1466 bp after removal of the repetitive portion of the control region. We generated a time‐calibrated phylogeny of these sequences using the root age calibration described above, assuming a constant population size through time and a HKY + G substitution model. All other parameters of the analysis were kept as described above.

## 3 RESULTS

Using targeted capture coupled with high throughput sequencing, we were able to recover near complete mitogenome sequences with ≥5× sequencing depth at each base for 32 samples, 15 from archival and 17 from non‐invasively collected samples (Table 1; Genbank accession numbers: OQ564458–OQ564489). Without considering alignment gaps and ambiguous base calls (i.e., Ns in sequences), we detected 25 unique haplotypes among the 32 mitogenomes. Putative haplotypes were mostly shared among sun bears from Cambodia (Table S5), however, one such putative haplotype was shared by bears from Thailand and Borneo (HMA_15_TH, HMA_19_BOR).

The 32 mitogenomes were analyzed together with three published sequences (GenBank: acc. no. MN807949, FM177765 and EF196664; see Table 1 for details). Summary statistics for the complete mitogenome dataset (*N =* 35), as well as subdivisions of the data based on phylogenetic analyses (below) are presented in Table 2.

**TABLE 2:** Summary statistics for sun bear mitogenome sequences.

Data	*N*	*h*	Hd	π	*S*	PIS
All	35	28	0.987	0.0068	419	245
Mainland clade	20	14	0.963	0.0017	162	52
Sunda clade	15	14	0.990	0.0034	221	97

*Note*: Number of samples (*N*), number of putative haplotypes (*h*), haplotype diversity (Hd), nucleotide diversity (π), segregating sites (*S*), parsimony informative sites (PIS). Estimates exclude sites with missing/ambiguous data or gaps.

### 3.1 Mitogenome phylogeny

**FIGURE 2:**
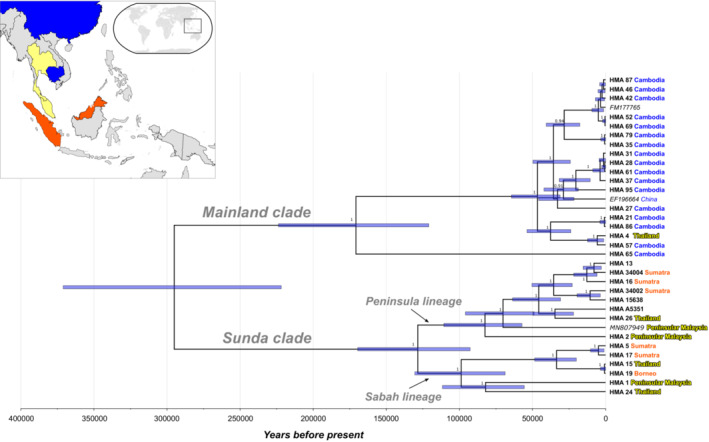
Bayesian phylogenetic tree reconstructed using *H. malayanus* mitogenome sequences (*N* = 35). Two main branches are labeled according to clade assignment, and previously reported lineages (Peninsula and Sabah; Lai, Chew, et al., 2021) are indicated. Sequences are labeled with sample ID and geographic origin (if known); text color of geographic origin follows the inset map (top left). For samples from Thailand, it is not known if they originate from North or South of the Isthmus of Kra. Blue bars indicate 95% credibility intervals for coalescence times in years.

Phylogenetic analysis of the sun bear mitogenome dataset (35 sequences) revealed two well‐supported clades (posterior clade credibility 1 for each clade), henceforth referred to as “Mainland clade” and “Sunda clade” following Kunde (2017), that diverged ~295 thousand years ago (kya; CI_95_: 371–221 kya; Figure 2). These clades are also present in the phylogeny reconstructed using a maximum likelihood (ML) approach (Figure S3).

The Mainland clade includes sequences (*N* = 20) from Cambodia, China, and Thailand, as well as a sequence from a sun bear of unknown provenance (Genbank acc. no. FM177765; Krause et al., 2008). Within this clade, two lineages diverged ~171 kya (CI_95_: 224–121 kya), of which one is currently only represented by a single sequence from Cambodia; support for this as a distinct lineage from the remaining mainland sequences is lower in the ML analysis (Figure S3). The remaining 19 sequences are in the second lineage, with a coalescence‐time estimated at ~47 kya (CI_95_: 64–31 kya). These sequences are mostly derived from Cambodian samples (16 of 19), and no obvious geographic structuring is apparent.

The Sunda clade includes sequences (*N* = 15) from Borneo, Sumatra and Peninsular Malaysia (all part of Sundaland), but also from Thailand, and from three archival samples of unknown provenance (sample details in Table S1). This clade also gave rise to two major lineages, which diverged ~128 kya (CI_95_: 170–93 kya), that correspond to mtDNA lineages previously identified (Lai, Chew, et al., 2021): the Peninsular lineage and Sabah lineage (indicated in Figure 2). These two lineages have a large geographic overlap: both include sun bears from Sumatra, Peninsular Malaysia and Thailand. Notably, we were only able to retrieve the mitogenome for one sample from Borneo, so we are unable to determine if both or only one of these lineages occurs on this island. Furthermore, as mentioned above, the sample from Borneo shared its putative haplotype with a sample from Thailand.

It is evident that both the Mainland and Sunda clades are present in Thailand (Figure 2; Figure S3). Unfortunately, it is not known whether these Thai sun bear samples originated from North or South of the Isthmus of Kra (5–13°N), an important zoogeographic transition zone between Sundaland and continental mainland.

### 3.2 Shorter mtDNA sequences

**FIGURE 3:**
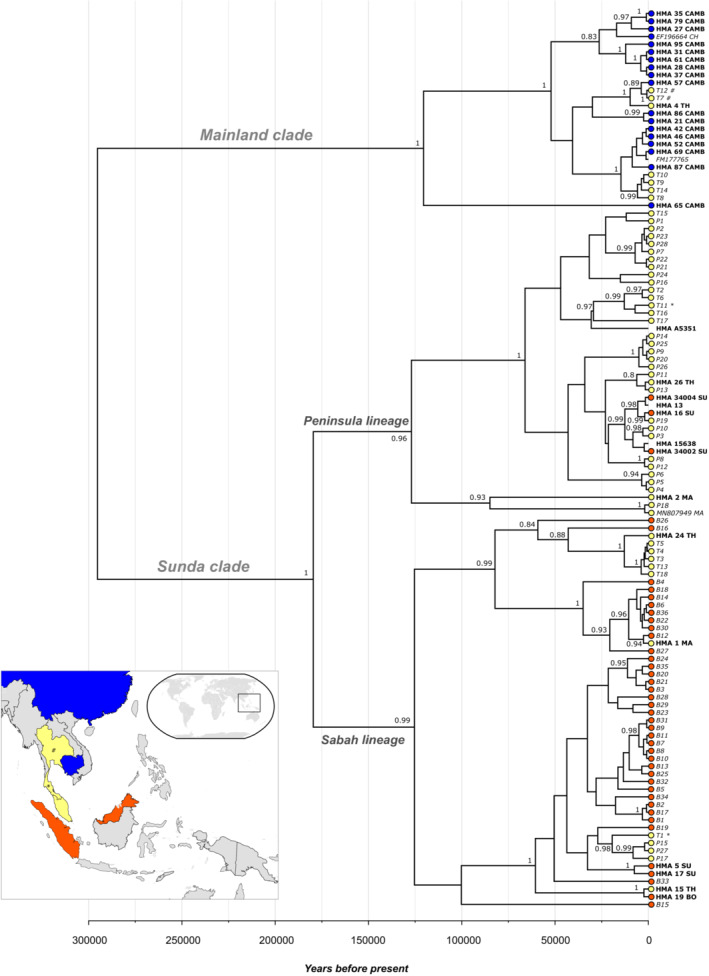
Bayesian phylogenetic tree reconstructed using a dataset of short mtDNA sequences (*N* = 117). Two main branches are labeled according to clade assignment, and previously reported lineages (Peninsula and Sabah; Lai, Chew, et al., 2021) are indicated. Sequences are labeled with sample ID; filled circles indicate if samples originate from China or Cambodia (blue), Thailand or Peninsular Malaysia (yellow), Sumatra or Borneo (orange), following the inset map (bottom left). For some samples from Thailand it is known if they originate from North or South of the Isthmus of Kra (IoK); this is indicated in sample ID and on the map using ‘#’ for North of the IoK and ‘*’ for South of the IoK.

We also considered the 35 mitogenome sequences in the context of a recent phylogenetic study on shorter mtDNA sequences of sun bears from Borneo, Peninsular Malaysia and Thailand (Lai, Chew, et al., 2021). Phylogenetic analysis of this dataset (*N =* 117 sequences; alignment length 1466 bp; Figure 3) also recovered the two major clades with high support (posterior clade credibility 1 for each clade; Figure 2), but both were much younger than the previous estimate of ~1.5 Mya (Lai, Chew, et al., 2021). This discrepancy in coalescence time estimates between the two studies likely reflects (i) our removal of the repetitive portion of the control region (which is difficult to resolve using short‐read data), and (ii) the differences in choice of calibration points and dating methods between the two studies (see Section 2 for details).

We also enlarged the Mainland clade by Thailand samples T7, T8, T9, T10, T12 and T14 (Lai, Chew, et al., 2021), bringing the total to 26 sequences. Consistent with the mitogenome phylogeny, there are two lineages in this clade, of which one is represented by a single sequence (HMA_65_CAMB), albeit with a more recent coalescence time ~121 kya (CI_95_: 181–66 kya).

Within the Sunda clade, we recovered the two previously identified major lineages: the Peninsula and the Sabah lineages (Lai, Chew, et al., 2021; indicated in Figure 3). Our estimate for the coalescence time of these lineages was ~180 kya (CI_95_: 260–112 kya), again, much later than the previous estimate of ~600 kya (Lai, Chew, et al., 2021), but earlier than the estimate of ~128 kya based on mitogenome data (Figure 2).

The Peninsula lineage (*N* = 40) consisted mostly of samples from Peninsular Malaysia (*N* = 27), but also included samples originating from Thailand (*N* = 7) and Sumatra (*N* = 3), as well as three samples of unknown origin (Figure 3). The coalescence time estimate for this lineage was ~127 kya (CI_95_: 193–73 kya), earlier than the estimate of ~82 kya based on mitogenome data (Figure 2). Consistent with the mitogenome phylogeny, this analysis revealed that the Peninsula lineage was also present in Sumatra. Our study also increased the number of sequences from Thailand and Peninsular Malaysia in this lineage.

The Sabah lineage (*N* = 51) consisted mostly of samples from Borneo (*N* = 37), but also included samples originating from Thailand (*N* = 8), Peninsular Malaysia (*N* = 4), and Sumatra (*N* = 2). The coalescence time estimate for this lineage was ~125 kya (CI_95_: 186–73 kya), older than the estimate of ~99 kya based on mitogenome data (Figure 2). Consistent with the mitogenome phylogeny, this analysis revealed that the Sabah lineage was also present in Sumatra. Our study also increased the number of sequences from Borneo, Thailand, and Peninsular Malaysia in this lineage.

Thus, both the Peninsula and Sabah lineages were found in Peninsular Malaysia, Thailand and Sumatra, while the Sabah lineage was also present on Borneo. Inclusion of our samples in this analysis reduced the geographic structuring suggested by the results of Lai, Chew, et al. (2021); that is to say, some sub‐lineages that were previously restricted to one geographic region, now include sequences from one or two additional geographic regions.

There is geographic origin information for some of the Thai sun bear samples from Lai, Chew, et al. (2021). Among the seven Thailand samples in the Mainland clade, there is geographic origin information for two (T7, T12), both of which come from North of the Isthmus of Kra (‘Thailand East’ in Lai, Chew, et al., 2021; indicated by ‘#’ in Figure 3). Among the 15 Thailand samples in the Sunda clade, there is geographic origin information for two (T1 in Sabah lineage, T11 in Peninsula lineage), both of which come from South of the Isthmus of Kra (‘Thailand West’ in Lai, Chew, et al., 2021; indicated by ‘*’ in Figure 3).

## 4 DISCUSSION

By incorporating archival and non‐invasively collected material in our sampling, and using targeted capture coupled with high throughput sequencing, we successfully retrieved data for mitochondrial genomes of sun bears from a large portion of their geographic distribution.

We identified two matrilines within the sampled distribution of the species: one restricted to portions of mainland SE Asia (encompassing China, Cambodia and Thailand), while the other occurs in Sundaland (Peninsular Malaysia, Sumatra and Borneo), as well as parts of the mainland (Thailand). Despite our efforts, the failure of some archival samples to yield results means that our coverage of the sun bear distribution in mainland SE Asia is unfortunately limited; we are missing data for Vietnam, Laos, Myanmar, and India.

It has been suggested that sun bears have originated in what is now Peninsular Malaysia and Borneo, and that the divergence of observed sun bear morphological forms commenced in the Pliocene (Meijaard, 2004). The coalescence time for the two clades indicates that sun bear populations diverged during the Middle Pleistocene, a much younger date than the proposed Pliocene divergence. Fossil evidence places sun bears in Indochina during the Middle Pleistocene (Louys, 2012), while evidence for the species' presence on the Sundaic islands is dated to the late Pleistocene (Long et al., 1996; Medway, 1964; Tougard, 2001), although an earlier presence is conceivable as the mammalian fossil record for Sumatra and Borneo is sparse prior to the Late Pleistocene (Meijaard, 2004). While molecular estimates of divergence times need to be considered with caution, our mitogenome data do suggest the presence of sun bears on the Sundaic islands by the late Middle Pleistocene.

It could be argued that the coalescence time for Sundaic sun bears ~128 kya is not consistent with the origin of sun bears in this region (i.e., Peninsular Malaysia and Borneo, following Meijaard, 2004), but this estimate only takes into account mitochondrial variation of extant populations in that region. It should also be noted that without the retrieval of the divergent mitogenome lineage within the Mainland clade from sample HMA_65_CAMB (Figure 2), the coalescence time estimate for sun bears of the Mainland clade would have been only ~47 kya, much younger than the Sundaic expansion. Further sampling throughout the sun bear distribution may improve our understanding of the species' origin, as additional mitogenome lineages may be unsampled thus far (e.g., Mengüllüoğlu et al., 2021). However, while the reconstructed mitochondrial phylogeny in general is very recent, historical and recent demographic processes may have resulted in loss of variation (mitochondrial lineages) that existed in the past, thereby obscuring older evolutionary processes. Thus, the question of the sun bears' origin is likely best addressed using nuclear markers. The issue is further compounded by possible secondary contact of sun bears in the region where the species may have originated.

### 4.1 Secondary contact

The geographic distribution of mitogenome clades reflects vicariance between sun bears of mainland Indochina and Sundaland, a biogeographic pattern that is observed in many other SE Asian species that are widely distributed (Woodruff & Turner, 2009). It is unknown if the Mainland clade was lost in sun bears before, during, or after colonization of the Sundaland. If this loss occurred before or during colonization, this suggests that sun bears from the Sunda clade have persisted in the Thai‐Malay Peninsula since the Middle Pleistocene. If the loss of the Mainland clade occurred after colonization, this suggests secondary contact of the two lineages in the Thai‐Malay Peninsula, as both major clades are found in Thailand. Such migration was possible when the exposed shelf connected the mainland and the major islands of the Sundaland during the low sea levels of the late Pleistocene (Bird et al., 2005), and has been inferred for sun bears (Lai, Chew, et al., 2021) and other large mammals, such as leopard *Panthera pardus* (Wilting et al., 2016), leopard cat *Prionailurus bengalensis* (Patel et al., 2017), and muntjac *Muntiacus muntjak* (Martins, Fickel, et al., 2017).

If there was secondary contact of these two lineages following a northward migration of sun bears of the Sunda clade, we can consider two scenarios: (1) the replacement of sun bears of the Mainland clade by bears of the Sunda clade in portions of the Thai‐Malay Peninsula, or (2) the recolonization of the Thai‐Malay Peninsula by bears from the Sunda clade following a local extinction of Mainland clade sun bears, possibly resulting from the Toba volcano super eruption on Sumatra ~74 kya (Costa et al., 2014). The first scenario suggests that Sundaic sun bears would have had some competitive advantage over individuals of the local mainland population during the period(s) when the Sunda Shelf was exposed, and migration of Sundaic bears to the Thai‐Malay Peninsula was possible. In the second scenario, we would need to consider that the volcanic eruption would have most likely severely affected the Sumatran sun bear population (as suggested for other species, e.g. leopard: Wilting et al., 2016). In this context, it is of note that the Peninsula lineage has thus far only been detected in Sumatra, Peninsular Malaysia and Thailand, but not on Borneo, raising the question where this lineage may have endured following the Toba eruption. A candidate region is Kalimantan, the Indonesian portion of Borneo, for which no mtDNA data are currently available.

Clearly, the available molecular data are not adequate to address these open questions, and further sampling is needed. Analyses should ideally also include nuclear genomic data, which would also be extremely valuable to address the taxonomic uncertainty regarding the sun bears of Borneo.

### 4.2 Bornean sun bears

There are clear phenotypic differences between the sun bears on Borneo and those from elsewhere in the species' range, and currently two subspecies are recognized: the broadly distributed *H. m. malayanus* and the Bornean *H. m. euryspilus* (Meijaard, 2004). However, several authors have suggested that further morphological and molecular data are needed to conclusively resolve the subspecific status of Bornean sun bears (Kitchener, 2010; Lai, Chew, et al., 2021).

Mitochondrial data can not disclose the mechanisms responsible for the observed phenotypic differences. However, it does show that Bornean sun bears harbor one Sundaic lineage (Sabah lineage) that is present throughout the region, including Sumatra, Peninsular Malaysia and Thailand, while a second widespread Sundaic lineage (Peninsula lineage) has thus far not been observed on Borneo. If the absence of the Peninsula lineage on Borneo is real (and not the result of sampling bias), then the question arises why the two lineages do not coexist on Borneo. It is also unknown how far their divergence time (~128 kya) relates to the divergence of Bornean sun bears. In this context, it can also not be discounted that historical or modern human‐mediated translocation of sun bears may have contributed to the observed distribution of mitochondrial lineages in the Sundaic region, further complicating matters.

### 4.3 Human‐mediated translocations

Sun bears are hunted for the illegal pet trade (Foley et al., 2011; Gomez et al., 2020; Krishnasamy & Shepherd, 2014; Lee et al., 2015; Shepherd & Shepherd, 2010), and are traded on and among the Sundaic islands (e.g., Gomez et al., 2019). Rescue and release of such trafficked bears may result in the introduction of non‐endemic mtDNA haplotypes into local populations. It is worth noting that such translocations do not need to be recent. There is a history of human‐mediated introduction or translocation of mammals (e.g., Long, 2003) for various reasons (e.g., cultural, commercial, pest‐control, accidental). Such translocations included large mammals and carnivores in SE Asia, among others cervids (*Rusa timorensis* and *R. unicolor*, Martins, Schmidt, et al., 2017), pigs (Groves, 1984), the leopard cat (*Prionailurus bengalensis*, Patel et al., 2017), the Malay civet (*Viverra tangalunga*, Veron et al., 2014), and the Asian palm civet (*Paradoxurus hermaphroditus*, Flannery et al., 1995). Because some of these human‐mediated translocations had commercial reasons (e.g., Groves, 1984), it is conceivable that there was also historical trade in bears and their derivatives (e.g., Hose, 1893), which may have included the translocation and subsequent (intentional or unintentional) release of sun bears. Investigation of Early Holocene samples using ancient DNA techniques could address the impact of human‐mediated translocations. Unfortunately, sun bear fossils are rare, and the species inhabits an environment that is not conducive to ancient DNA preservation (e.g., Paijmans et al., 2013).

## 5 CONCLUDING REMARKS

Thus far, the classification of sun bear populations has been based on geography and morphological traits, the latter having only been investigated using a small dataset. It is important to corroborate/supplement such efforts using molecular studies (Kitchener, 2010). Our study reveals a clear and deep split into two matrilines that divide sun bears from the Sundaic region (Borneo, Sumatra, Peninsular Malaysia, Thailand) from others on the Indochinese mainland (China, Cambodia, Thailand). As has been observed in mtDNA studies of other SE Asian vertebrates, these Sundaic and mainland lineages are both present in the Thai‐Malay Peninsula. With regards to sun bear taxonomy, we observe that the Bornean subspecies *H. m. euryspilus* does not carry a distinctive mitochondrial lineage, but rather one that is present throughout Sundaland (Borneo, Sumatra, Peninsular Malaysia, Thailand). To improve our understanding of sun bear evolution and biogeography, further molecular studies are needed. Two obvious priorities are to include samples from a border geographical context and to obtain data from nuclear genomes. Until such research has been carried out, we encourage ex‐situ conservation management to take the existence of the two highly differentiated matrilines into account.

## AUTHOR CONTRIBUTIONS

**Miriam N. Kunde:** Conceptualization (equal); data curation (lead); formal analysis (equal); investigation (equal); project administration (lead); resources (equal); writing – original draft (lead); writing – review and editing (equal). **Axel Barlow:** Data curation (supporting); formal analysis (equal); investigation (equal); software (supporting); visualization (supporting); writing – review and editing (supporting). **Achim M. Klittich:** Data curation (supporting); formal analysis (supporting); investigation (supporting); software (supporting); visualization (supporting); writing – review and editing (supporting). **Aliya Yakupova:** Data curation (supporting); formal analysis (supporting); investigation (supporting); resources (supporting); software (supporting); writing – review and editing (supporting). **Riddhi P. Patel:** Data curation (supporting); formal analysis (supporting); investigation (supporting); methodology (supporting); software (supporting); supervision (supporting); writing – review and editing (supporting). **Jorns Fickel:** Conceptualization (supporting); funding acquisition (supporting); project administration (supporting); supervision (supporting); writing – review and editing (supporting). **Daniel W. Förster:** Conceptualization (lead); data curation (supporting); formal analysis (equal); funding acquisition (lead); investigation (supporting); methodology (lead); project administration (supporting); resources (supporting); software (supporting); supervision (lead); validation (equal); visualization (supporting); writing – original draft (supporting); writing – review and editing (supporting).

## FUNDING INFORMATION

This work was funded by the Leibniz‐Association grant SAW‐2013‐IZW‐2.

## CONFLICT OF INTEREST STATEMENT

None.

## Supporting information


Appendix S1

## Data Availability

We have deposited the primary data underlying these analyses as follows: Mitochondrial genome sequences: GenBank acc. no. OQ564458–OQ564489; Sampling locations, haplotypes, and collection details are in Table S1; Cytochrome B primer sequences to verify target species are in Table S2; Sequences used in analysis of short mtDNA sequence are in Table S4.
