# CORRIGENDUM

**DOI:** 10.1002/ece3.10283

**Published:** 2023-07-09

In the recent article by Kunde et al. (2023), the authors would like to add the following funding information:

Open Access funding enabled and organized by Projekt DEAL. The publication of this article was funded by the Deutsche Forschungsgemeinschaft (DFG, German Research Foundation) – project number 491292795.

The article has been corrected online.

## References

[ece310283-bib-0001] Kunde, M. N. , Barlow, A. , Klittich, A. M. , Yakupova, A. , Patel, R. P. , Fickel, J. , & Förster, D. W. (2023). First mitogenome phylogeny of the sun bear *Helarctos malayanus* reveals a deep split between Indochinese and Sundaic lineages. Ecology and Evolution, 13, e9969. 10.1002/ece3.9969 37082317 PMC10111171

