# Supplementary material for: First mitogenome phylogeny of the sun bear Helarctos malayanus reveals a deep split between Indochinese and Sundaic lineages
Source: Ecol Evol. 2023 Apr 18;13(4):e9969. doi: 10.1002/ece3.9969 (PMC10111171; doi:10.1002/ece3.9969)

Online Supplemental Material for

**First mitogenome phylogeny of the sun bear *Helarctos malayanus* reveals a deep split between Indochinese and Sundaic lineages**

Miriam N. Kunde, Axel Barlow, Achim M. Klittich, Aliya Yakupova, Riddhi P. Patel, Jörns Fickel, Daniel W. Förster

| Contents |  |
| --- | --- |
| Suppl. Table S1: detailed sample info | Pg. 1-4 |
| Suppl. Table S2: cytochrome b primers & PCR details | Pg. 4 |
| Suppl. Table S3: Substitution models (BEAST) | Pg. 5 |
| Suppl. Table S4: sample details for analysis of short mtDNA sequences | Pg. 6-8 |
| Suppl. Table S5: shared mitogenome haplotypes | Pg. 8 |
| Suppl. Figure S1: Species level analysis of Ursidae based on mitochondrial genomes | Pg. 9 |
| Suppl. Figure S2: Maximum-likelihood tree for species level analysis, based on mitochondrial genomes. | Pg. 10 |
| Suppl. Figure S3: Maximum-likelihood tree based on sun bear mitochondrial sequences | Pg. 11 |

**Suppl. Table S1**: detailed sample info

| **Sample ID** | **Collection Location** | **Museum or  Sanctuary  Location** | **Mueum/**  **Sanctuary** | **Museum or Sanctuary ID** | **collection year** | **Provided by** | **Sample type** |
| --- | --- | --- | --- | --- | --- | --- | --- |
| HMA_1_MA | Jahore, Malaysia | Stuttgart, Germany | SMNS | 21754 | 1897 | Dr. Stefan Merker, Staatliches Museum für Naturkunde Stuttgart | nasal bone |
| HMA_2_MA | Jahore, Malaysia | Stuttgart, Germany | SMNS | 21753 | 1897 | Dr. Stefan Merker, Staatliches Museum für Naturkunde Stuttgart | nasal bone |
| HMA_3_IN | Burma/Vorderasien | Bonn, Germany | ZFMK | ZFMK 32.21 | *unknown* | Dr. Jan Decher, Zoologisches Forschungsinstitut und Museum Alexander Koenig, Bonn | skin |
| HMA_4_TH | Siam (Thailand) | Bonn, Germany | ZFMK | ZFMK 81.55 | 1921 | Dr. Jan Decher, Zoologisches Forschungsinstitut und Museum Alexander Koenig, Bonn | tissue |
| HMA_5_SU | prov. Deli, Nord-Sumatra | Bonn, Germany | ZFMK | ZFMK 65.5 | 1963/64 | Dr. Jan Decher, Zoologisches Forschungsinstitut und Museum Alexander Koenig, Bonn | bone (vertebra) |
| HMA_6_JA | Java, Indonesia | Wien, Austria | NHM | B 4249 | Oct, 1929 | Dr. habil. Frank Emmanuel Zachos, Naturhistorisches Museum Wien | Skin (fur) |
| HMA_7_SU | Sumatra | Dresden, Germany | MTD | B6076 | 1827 | Dr. Clara Stefen, Senckenberg Naturhistorische Sammlungen Dresden | Skin+ hair |
| HMA_8_VI | Vietnam | Paris, France | MNHN | 1929-430 | *unknown* | Geraldine Veron, Museum National d'Histoire Naturelle, Paris | tissue from skull |
| HMA_9_JA | Java, Indonesia | Paris, France | MNHN | A-2132 | *unknown* | Geraldine Veron, Museum National d'Histoire Naturelle, Paris | tissue from skull |
| HMA_10_CH | Ho-Chi-Minh-city/Saigon | Frankfurt, Germany | SNG | 5790 | *unknown* | Katrin Krohmann, Senckenberg Naturkundemuseum, Frankfurt | skin |
| HMA_11_SU | Sunda Islands, SE-Asia | Frankfurt, Germany | SNG | 15461 | *unknown* | Katrin Krohmann, Senckenberg Naturkundemuseum, Frankfurt | skin |
| HMA_12_SU | Sunda Islands, SE-Asia | Frankfurt, Germany | SNG | 15776 | *unknown* | Katrin Krohmann, Senckenberg Naturkundemuseum, Frankfurt | skin |
| HMA_13 | Zoo animal | Frankfurt, Germany | SNG | 15777 | *unknown* | Katrin Krohmann, Senckenberg Naturkundemuseum, Frankfurt | dry tissue from skin |
| HMA_14_SU | Lampong, South-Sumatra | Munich, Germany | ZSM | 1908/539 | *unknown* | Michael Hiermeier, Zoologische Staatssammlung München | loose bones (tissue) |
| HMA_15_TH | Siam (Thailand) | Munich, Germany | ZSM | 1918/31 | *unknown* | Michael Hiermeier, Zoologische Staatssammlung München | Skeleton (tissue) |
| HMA_16_SU | Medan Deli, east coast Sumatra | Munich, Germany | ZSM | 1918/482 | *unknown* | Michael Hiermeier, Zoologische Staatssammlung München | Skull (nasal bone) |
| HMA_17_SU | Medan Deli, east coast Sumatra | Munich, Germany | ZSM | 1910/154 | *unknown* | Michael Hiermeier, Zoologische Staatssammlung München | Skull (nasal bone) |
| HMA_18_BO | Borneo | Munich, Germany | ZSM | 1907/231 | *unknown* | Michael Hiermeier, Zoologische Staatssammlung München | Skull (nasal bone) |
| HMA_19_BO | Borneo | Munich, Germany | ZSM | 1907/485 | *unknown* | Michael Hiermeier, Zoologische Staatssammlung München | Skull (nasal bone) |
| HMA_20_BO | Borneo | Munich, Germany | ZSM | 1907/471 | *unknown* | Michael Hiermeier, Zoologische Staatssammlung München | Skull (nasal bone) |
| HMA_21_BO | Borneo | Munich, Germany | ZSM | 1907/638 | *unknown* | Michael Hiermeier, Zoologische Staatssammlung München | Skull (nasal bone) |
| HMA_22_TH | Siam (Thailand) | Munich, Germany | ZSM | 1904/169 | May-04 | Michael Hiermeier, Zoologische Staatssammlung München | Skull (nasal bone) |
| HMA_23_TH | Siam (Thailand) | Munich, Germany | ZSM | 1906/143 | *unknown* | Michael Hiermeier, Zoologische Staatssammlung München | Skull (nasal bone) |
| HMA_24_TH | Siam (Thailand) | Munich, Germany | ZSM | 1906/142 | *unknown* | Michael Hiermeier, Zoologische Staatssammlung München | Skull (nasal bone) |
| HMA_25_TH | Siam (Thailand) | Munich, Germany | ZSM | 1911/19 | 09/03/1911 | Michael Hiermeier, Zoologische Staatssammlung München | Skull (nasal bone) |
| HMA_26_TH | Siam (Thailand) | Munich, Germany | ZSM | 1906/149 | *unknown* | Michael Hiermeier, Zoologische Staatssammlung München | Skull (nasal bone) |
| HMA_13_CAMB | Siem Reap (Cambodia) | Pnom Penh, Cambodia | FtB | Ranee | 2014 | Free the Bears/ Kingdom of Cambodia | Saliva |
| HMA_28_CAMB | Kompong Cham (Cambodia) | Pnom Penh, Cambodia | FtB | Mien | 2014 | Free the Bears/ Kingdom of Cambodia | Saliva |
| HMA_35_CAMB | Siem Reap (Cambodia) | Pnom Penh, Cambodia | FtB | Tonle Sap | 2014 | Free the Bears/ Kingdom of Cambodia | Saliva |
| HMA_42_CAMB | Koh Kong (Cambodia) | Pnom Penh, Cambodia | FtB | Kong | 2014 | Free the Bears/ Kingdom of Cambodia | Saliva |
| HMA_57_CAMB | Koh Kong (Cambodia) | Pnom Penh, Cambodia | FtB | Ooshie | 2014 | Free the Bears/ Kingdom of Cambodia | Saliva |
| HMA_79_CAMB | Oddar Meanchey (Cambodia) | Pnom Penh, Cambodia | FtB | Sam | 2014 | Free the Bears/ Kingdom of Cambodia | Saliva |
| HMA_86_CAMB | Prey Vihear (Cambodia) | Pnom Penh, Cambodia | FtB | Win | 2014 | Free the Bears/ Kingdom of Cambodia | Saliva |
| HMA_31_CAMB | Oddar Meanchey (Cambodia) | Pnom Penh, Cambodia | FtB | Phat | 2014 | Free the Bears/ Kingdom of Cambodia | Saliva |
| HMA_52_CAMB | Kompong Speu (Cambodia) | Pnom Penh, Cambodia | FtB | Hasa | 2014 | Free the Bears/ Kingdom of Cambodia | Saliva |
| HMA_61_CAMB | Preah Vihear (Cambodia) | Pnom Penh, Cambodia | FtB | Hope-bee | 2014 | Free the Bears/ Kingdom of Cambodia | Saliva |
| HMA_65_CAMB | Preah Vihear (Cambodia) | Pnom Penh, Cambodia | FtB | Jacko | 2014 | Free the Bears/ Kingdom of Cambodia | Saliva |
| HMA_80_CAMB | Kratie (Cambodia) | Pnom Penh, Cambodia | FtB | Kratie | 2014 | Free the Bears/ Kingdom of Cambodia | Saliva |
| HMA_90_CAMB | Stung Treng (Cambodia) | Pnom Penh, Cambodia | FtB | Sybil Sunbeam | 2014 | Free the Bears/ Kingdom of Cambodia | Saliva |
| HMA_21_CAMB | Kompong Thom (Cambodia) | Pnom Penh, Cambodia | FtB | Ju-ju | 2014 | Free the Bears/ Kingdom of Cambodia | Saliva |
| HMA_27_CAMB | Kratie (Cambodia) | Pnom Penh, Cambodia | FtB | Dodo | 2014 | Free the Bears/ Kingdom of Cambodia | Saliva |
| HMA_37_CAMB | Ratanakiri (Cambodia) | Pnom Penh, Cambodia | FtB | Buddy | 2014 | Free the Bears/ Kingdom of Cambodia | Saliva |
| HMA_46_CAMB | Kompong Thom (Cambodia) | Pnom Penh, Cambodia | FtB | Kiem | 2014 | Free the Bears/ Kingdom of Cambodia | Saliva |
| HMA_87_CAMB | Kompong Som (Cambodia) | Pnom Penh, Cambodia | FtB | Deena | 2014 | Free the Bears/ Kingdom of Cambodia | Saliva |
| HMA_88_CAMB | Ratanakiri (Cambodia) | Pnom Penh, Cambodia | FtB | Abbie | 2014 | Free the Bears/ Kingdom of Cambodia | Saliva |
| HMA_95_CAMB | Pursat (Cambodia) | Pnom Penh, Cambodia | FtB | Holly | 2014 | Free the Bears/ Kingdom of Cambodia | Saliva |
| HMA_69_CAMB | Stung Treng (Cambodia) | Pnom Penh, Cambodia | FtB | Po Sary | 2014 | Free the Bears/ Kingdom of Cambodia | Saliva |
| HMA_34002_SU | Sumatra | Berlin, Germany | ZMB | ZMB_MA_34002 | *unknown* | Dr. Frieder Mayer, Naturkundemuseum Berlin | Skull |
| HMA_34004_SU | Sumatra | Berlin, Germany | ZMB | ZMB_MA_34004 | *unknown* | Dr. Frieder Mayer, Naturkundemuseum Berlin | Skull |
| HMA_17531_TH | Thailand | Berlin, Germany | ZMB | ZMB_MA_17531 | *unknown* | Dr. Frieder Mayer, Naturkundemuseum Berlin | Skull |
| HMA_15638 | *unknown* | Berlin, Germany | ZMB | ZMB_MA_15638 | *unknown* | Dr. Frieder Mayer, Naturkundemuseum Berlin | Skull |
| HMA_A5351 | *unknown* | Berlin, Germany | ZMB | ZMB_MA_A5351 | *unknown* | Dr. Frieder Mayer, Naturkundemuseum Berlin | Skull |
| HMA_17245_BO | Borneo | Berlin, Germany | ZMB | ZMB_MA_17245 | *unknown* | Dr. Frieder Mayer, Naturkundemuseum Berlin | Skull |

**Suppl. Table S2**: cytochrome b primers & PCR details

| **Primers** | F: ACACCGAAATCTTTCTCACT  R: AAGGAAATAAAATGCTCGGAGAC |
| --- | --- |
| **PCR mix** | Reaction volume of 15 µl:  6.48 µl of dH2O, 3 µl GoTaq Buffer, 1.2 µl MgCl2 (25 mM), 1 µl BSA (20mM), 0.25 µl dNTPs (10mM), 0.5 µl Primer F (10mM), 0.5 µl Primer R (10mM), 0.075 µl GoTaq Polym (5 U/ µl), 2 µl DNA template |
| **Thermocycler conditions** | Initial denaturation at  95° C for 2 min, then 40 cycles of denaturion at 95° C for 30 s, annealing at 59° C for 30 s, and extension at 72° C for 30 s, with a final extension at 72° C for 5 min. |

**Suppl. Table S3:** Substitution models (BEAST)

*Species Level Analysis*

| **Partition** | **BIC Model** | **Used model** | **Nsites** | **mtDNA regions** |
| --- | --- | --- | --- | --- |
| 1 | TRN+I+G+X | TRN+I+G+X | 5246 | Leu2, Tyr, Lys, Asp, Ile, Arg, His, Ser2, Asn, ND6_CP2, Ala, ND2_CP1, ND4_CP1, ND5_CP1, Trp, Gln, Gly, Glu, 16s_p1, 12s, 16s_pt2, Cys |
| 2 | K80+G | K80+G | 1292 | COX2_CP1, ND1_CP1, Ser1, Leu1, ND4L_CP1, ND3_CP1, CYTB_CP1 |
| 3 | HKY+I+G+X | HKY+I+G+X | 2724 | CYTB_CP2, ND2_CP2, ND3_CP2, ND4L_CP2, COX1_CP2, COX3_CP2, COX2_CP2, ND1_CP2, ND4_CP2 |
| 4 | TRN+I+G+X | TRN+I+G+X | 2757 | ND2_CP3, ND4_CP3, ND3_CP3, COX3_CP3, CYTB_CP3, ND6_CP3, ND4L_CP3, ND1_CP3, ND5_CP3 |
| 5 | K80+I | K80+I | 848 | Met, COX1_CP1, COX3_CP1 |
| 6 | HKY+G+X | HKY+G+X | 1023 | ATP8_ATP6_CP2, COX2_CP3, COX1_CP3 |
| 7 | TRN+I+G+X | TRN+G+X | 1130 | ND6_CP1, Thr, ATP8_ATP6_CP1, ND5_CP2 |
| 8 | HKY+G+X | HKY+G+X | 1448 | D-loop, Val, Pro, ATP8_ATP6_CP3, Phe |
|  |  |  |  |  |

*Population Level Analysis*

| **Partition** | **BIC Model** | **Used model** | **Nsites** | **mtDNA regions** |
| --- | --- | --- | --- | --- |
| 1 | K80 | K80 | 1681 | Met, Trp, 16s_pt2, Cys, ND4L_CP1, COX3_CP1, COX1_CP1, COX2_CP1 |
| 2 | HKY+G+X | HKY+G+X | 2181 | D-loop, Leu1, Pro, Gly, COX2_CP3, COX1_CP3, ATP8_ATP6_CP2 |
| 3 | K80+I | K80+I | 815 | CYTB_CP1, ND3_CP1, ND1_CP1 |
| 4 | HKY+X | HKY+X | 3676 | Tyr, COX2_CP2, COX3_CP2, COX1_CP2, CYTB_CP2, ND5_CP2, ATP8_ATP6_CP1, ND4L_CP2, ND3_CP2, ND2_CP2, ND4_CP2, ND1_CP2 |
| 5 | HKY+X | HKY+X | 2933 | ND6_CP1, CYTB_CP3, ND6_CP3, ND4L_CP3, ND2_CP3, ND1_CP3, ND5_CP3, COX3_CP3, ND3_CP3, ND4_CP3 |
| 6 | HKY+X | HKY+X | 425 | Leu2, Lys, Arg, Ile, His, Asp |
| 7 | TRN+X | TRN+X | 4757 | Ser2, Asn, Ser1, Thr, Ala, Gln, Val, Phe, Glu, ATP8_ATP6_CP3, ND4_CP1, ND2_CP1, ND6_CP2, 16s_p1, 12s, ND5_CP1 |

**Suppl. Table S4:** sample details for analysis of short mtDNA sequences

| **Sample ID** | **Region** | **Sample Type** | **Accession Number** | **Study** |
| --- | --- | --- | --- | --- |
| HMA_1_MA | Peninsular Malaysia | archival | OQ564458 | *this study* |
| HMA_2_MA | Peninsular Malaysia | archival | OQ564459 | *this study* |
| HMA_4_TH | Thailand | archival | OQ564460 | *this study* |
| HMA_5_SU | Sumatra | archival | OQ564461 | *this study* |
| HMA_13 | *unknown* | archival | OQ564462 | *this study* |
| HMA_15_TH | Thailand | archival | OQ564463 | *this study* |
| HMA_16_SU | Sumatra | archival | OQ564464 | *this study* |
| HMA_17_SU | Sumatra | archival | OQ564465 | *this study* |
| HMA_19_BO | Borneo | archival | OQ564466 | *this study* |
| HMA_24_TH | Thailand | archival | OQ564468 | *this study* |
| HMA_26_TH | Thailand | archival | OQ564469 | *this study* |
| HMA_21_CAMB | Cambodia | saliva | OQ564467 | *this study* |
| HMA_27_CAMB | Cambodia | saliva | OQ564470 | *this study* |
| HMA_28_CAMB | Cambodia | saliva | OQ564471 | *this study* |
| HMA_31_CAMB | Cambodia | saliva | OQ564472 | *this study* |
| HMA_35_CAMB | Cambodia | saliva | OQ564473 | *this study* |
| HMA_37_CAMB | Cambodia | saliva | OQ564474 | *this study* |
| HMA_42_CAMB | Cambodia | saliva | OQ564475 | *this study* |
| HMA_46_CAMB | Cambodia | saliva | OQ564476 | *this study* |
| HMA_52_CAMB | Cambodia | saliva | OQ564477 | *this study* |
| HMA_57_CAMB | Cambodia | saliva | OQ564478 | *this study* |
| HMA_61_CAMB | Cambodia | saliva | OQ564479 | *this study* |
| HMA_65_CAMB | Cambodia | saliva | OQ564480 | *this study* |
| HMA_69_CAMB | Cambodia | saliva | OQ564481 | *this study* |
| HMA_79_CAMB | Cambodia | saliva | OQ564482 | *this study* |
| HMA_86_CAMB | Cambodia | saliva | OQ564483 | *this study* |
| HMA_87_CAMB | Cambodia | saliva | OQ564484 | *this study* |
| HMA_95_CAMB | Cambodia | saliva | OQ564485 | *this study* |
| HMA_34002_SU | Sumatra | archival | OQ564487 | *this study* |
| HMA_34004_SU | Sumatra | archival | OQ564488 | *this study* |
| HMA_15638 | *unknown* | archival | OQ564486 | *this study* |
| HMA_A5351 | *unknown* | archival | OQ564489 | *this study* |
| FM177765 | *unknown* | *-* | FM177765 | Krause et al. 2008 |
| EF196664 | China | *-* | EF196664 | Yu et al. 2007 |
| MN807949 | Peninsular Malaysia | *-* | MN807949 | Lai et al. 2021b |
| P1 | Peninsula/Johor | Blood on FTA card | MW316360 | Lai et al. 2021a |
| P2 | Peninsula/Pahang | Blood on FTA card | MW316361 | Lai et al. 2021a |
| P3 | Peninsula/Pahang | Blood on FTA card | MW316362 | Lai et al. 2021a |
| P4 | Peninsula/Perak | Blood on FTA card | MW316363 | Lai et al. 2021a |
| P5 | Peninsula/Perak | Blood on FTA card | MW316364 | Lai et al. 2021a |
| P6 | Peninsula/Selangor | Blood on FTA card | MW316365 | Lai et al. 2021a |
| P7 | Peninsula/- | Blood on FTA card | MW316366 | Lai et al. 2021a |
| P8 | Peninsula/Pahang | Blood on FTA card | MW316367 | Lai et al. 2021a |
| P9 | Peninsula/Pahang | Blood on FTA card | MW316368 | Lai et al. 2021a |
| P10 | Peninsula/Pahang | Incisor | MW316369 | Lai et al. 2021a |
| P11 | Peninsula/Penang | Hair | MW316370 | Lai et al. 2021a |
| P12 | Peninsula/Pahang | Hair | MW316371 | Lai et al. 2021a |
| P13 | Peninsula/Johor | Hair | MW316372 | Lai et al. 2021a |
| P14 | Peninsula/Perak | Hair | MW316373 | Lai et al. 2021a |
| P15 | Confiscated | Hair | MW316374 | Lai et al. 2021a |
| P16 | Peninsula/Kedah | Hair | MW316375 | Lai et al. 2021a |
| P17 | Confiscated | Hair | MW316376 | Lai et al. 2021a |
| P18 | Peninsula/- | Muscle | MW316377 | Lai et al. 2021a |
| P19 | Peninsula/Terengganu | Muscle | MW316378 | Lai et al. 2021a |
| P20 | Peninsula/Zoo | Hair | MW316379 | Lai et al. 2021a |
| P21 | Peninsula/Zoo | Hair | MW316380 | Lai et al. 2021a |
| P22 | Peninsula/Terengganu | Hair | MW316381 | Lai et al. 2021a |
| P23 | Peninsula/Zoo | Hair | MW316382 | Lai et al. 2021a |
| P24 | Peninsula/Pahang | Hair | MW316383 | Lai et al. 2021a |
| P25 | Peninsula/Selangor | Hair | MW316384 | Lai et al. 2021a |
| P26 | Peninsula/Melaka | Blood in EDTA | MW316385 | Lai et al. 2021a |
| P27 | Peninsula/Zoo | Blood in EDTA | MW316386 | Lai et al. 2021a |
| P28 | Peninsula/Zoo | Blood in EDTA | MW316387 | Lai et al. 2021a |
| B1 | Sabah/Keningau | Hair | MW316324 | Lai et al. 2021a |
| B2 | Sabah/— | Hair | MW316325 | Lai et al. 2021a |
| B3 | Sabah/Lahad Datu | Hair | MW316326 | Lai et al. 2021a |
| B4 | Sabah/Pitas | Hair | MW316327 | Lai et al. 2021a |
| B5 | Sabah/— | Hair | MW316328 | Lai et al. 2021a |
| B6 | Sabah/Tawau | Hair | MW316329 | Lai et al. 2021a |
| B7 | Sabah/Kota Kinabalu | Hair | MW316330 | Lai et al. 2021a |
| B8 | Sabah/Tawau | Hair | MW316331 | Lai et al. 2021a |
| B9 | Sabah/Sipitang | Hair | MW316332 | Lai et al. 2021a |
| B10 | Sabah/Penampang | Hair | MW316333 | Lai et al. 2021a |
| B11 | Sabah/Penampang | Hair | MW316334 | Lai et al. 2021a |
| B12 | Sabah/Ranau | Hair | MW316335 | Lai et al. 2021a |
| B13 | Sabah/Kuamut | Hair | MW316336 | Lai et al. 2021a |
| B14 | Sabah/Kudat | Hair | MW316337 | Lai et al. 2021a |
| B15 | Sabah/Kinabatangan | Hair | MW316338 | Lai et al. 2021a |
| B16 | Sabah/Ranau | Hair | MW316339 | Lai et al. 2021a |
| B17 | Sabah/— | Hair | MW316340 | Lai et al. 2021a |
| B18 | Sabah/— | Hair | MW316341 | Lai et al. 2021a |
| B19 | Sabah/Kota Marudu | Hair | MW316342 | Lai et al. 2021a |
| B20 | Sabah/Sipitang | Hair | MW316343 | Lai et al. 2021a |
| B21 | Sabah/Tawau | Hair | MW316344 | Lai et al. 2021a |
| B22 | Sabah/— | Hair | MW316345 | Lai et al. 2021a |
| B23 | Sabah/Kudat | Hair | MW316346 | Lai et al. 2021a |
| B24 | Sabah/Sipitang | Hair | MW316347 | Lai et al. 2021a |
| B25 | Sabah/Tawau | Hair | MW316348 | Lai et al. 2021a |
| B26 | Sabah/Tawau | Hair | MW316349 | Lai et al. 2021a |
| B27 | Sabah/Keningau | Hair | MW316350 | Lai et al. 2021a |
| B28 | Sabah/Labuk dan Sugut | Hair | MW316351 | Lai et al. 2021a |
| B29 | Sabah/— | Hair | MW316352 | Lai et al. 2021a |
| B30 | Sabah/Nabawan | Hair | MW316353 | Lai et al. 2021a |
| B31 | Sabah/— | Hair | MW316354 | Lai et al. 2021a |
| B32 | Sabah/Kota Marudu | Hair | MW316355 | Lai et al. 2021a |
| B33 | Sabah/Kudat | Hair | MW316356 | Lai et al. 2021a |
| B34 | Sabah/Labuk dan Sugut | Hair | MW316357 | Lai et al. 2021a |
| B35 | Sabah/Labuk dan Sugut | Hair | MW316358 | Lai et al. 2021a |
| B36 | Sabah/Tawau | Hair | MW316359 | Lai et al. 2021a |
| T1 | Thailand/West | Hair | MW316388 | Lai et al. 2021a |
| T2 | Thailand/— | Hair | MW316389 | Lai et al. 2021a |
| T3 | Thailand/— | Hair | MW316390 | Lai et al. 2021a |
| T4 | Thailand/— | Hair | MW316391 | Lai et al. 2021a |
| T5 | Thailand/— | Hair | MW316392 | Lai et al. 2021a |
| T6 | Thailand/— | Hair | MW316393 | Lai et al. 2021a |
| T7 | Thailand/East | Hair | MW316394 | Lai et al. 2021a |
| T8 | Thailand/— | Hair | MW316395 | Lai et al. 2021a |
| T9 | Thailand/— | Hair | MW316396 | Lai et al. 2021a |
| T10 | Thailand/— | Hair | MW316397 | Lai et al. 2021a |
| T11 | Thailand/West | Hair | MW316398 | Lai et al. 2021a |
| T12 | Thailand/East | Hair | MW316399 | Lai et al. 2021a |
| T13 | Thailand/— | Hair | MW316400 | Lai et al. 2021a |
| T14 | Thailand/— | Hair | MW316401 | Lai et al. 2021a |
| T15 | Thailand/— | Hair | MW316402 | Lai et al. 2021a |
| T16 | Thailand/— | Hair | MW316403 | Lai et al. 2021a |
| T17 | Thailand/— | Hair | MW316404 | Lai et al. 2021a |
| T18 | Thailand/— | Hair | MW316405 | Lai et al. 2021a |

**Suppl. Table S5**: shared mitogenome haplotypes

| HMA_52_CAMB, HMA_69_CAMB |
| --- |
| HMA_87_CAMB, HMA_46_CAMB |
| HMA_35_CAMB, HMA_79_CAMB |
| HMA_28_CAMB, HMA_31_CAMB, HMA_61_CAMB |
| HMA_86_CAMB, HMA_21_CAMB |
| HMA_15_TH, HMA_19_BO |

**Suppl. Figure S1**. Species level analysis of Ursidae based on mitochondrial genomes.


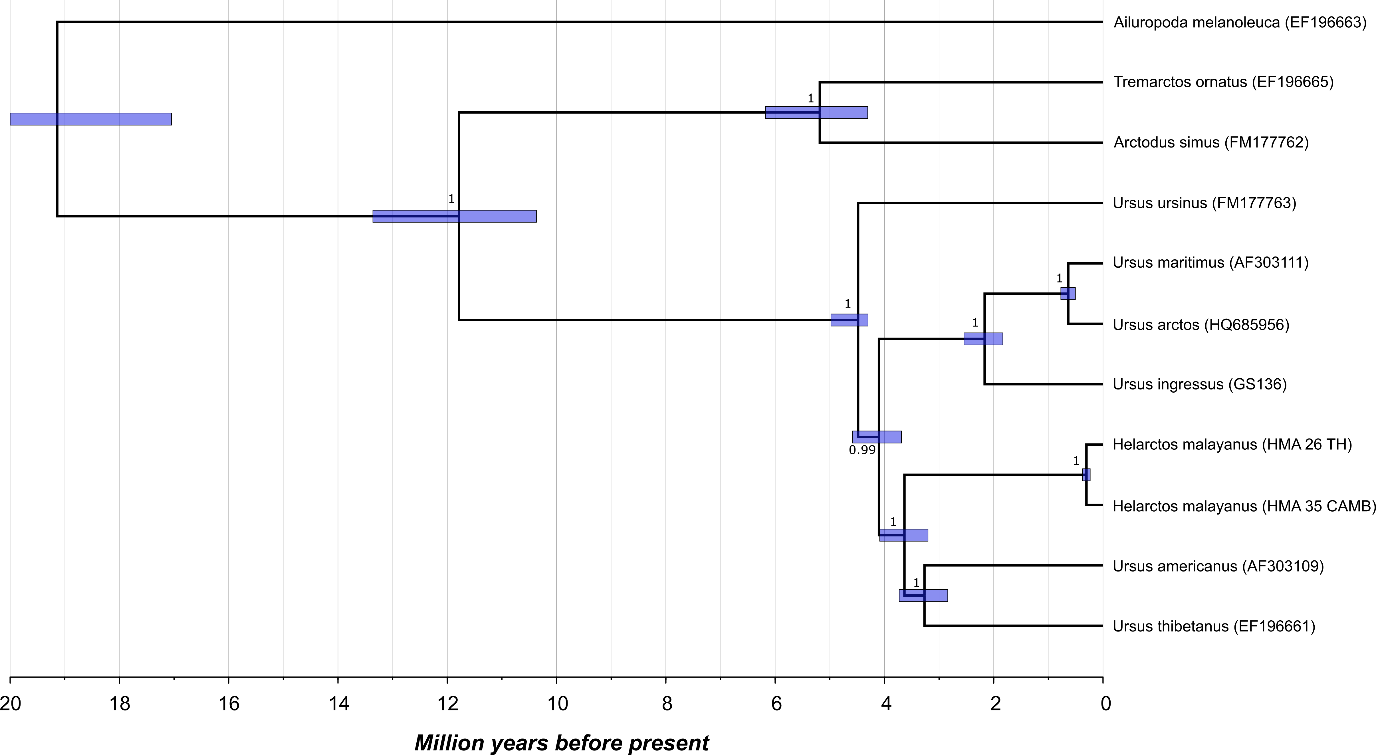


**Suppl. Figure S2**. Maximum-likelihood tree for species level analysis, based on mitochondrial genomes.


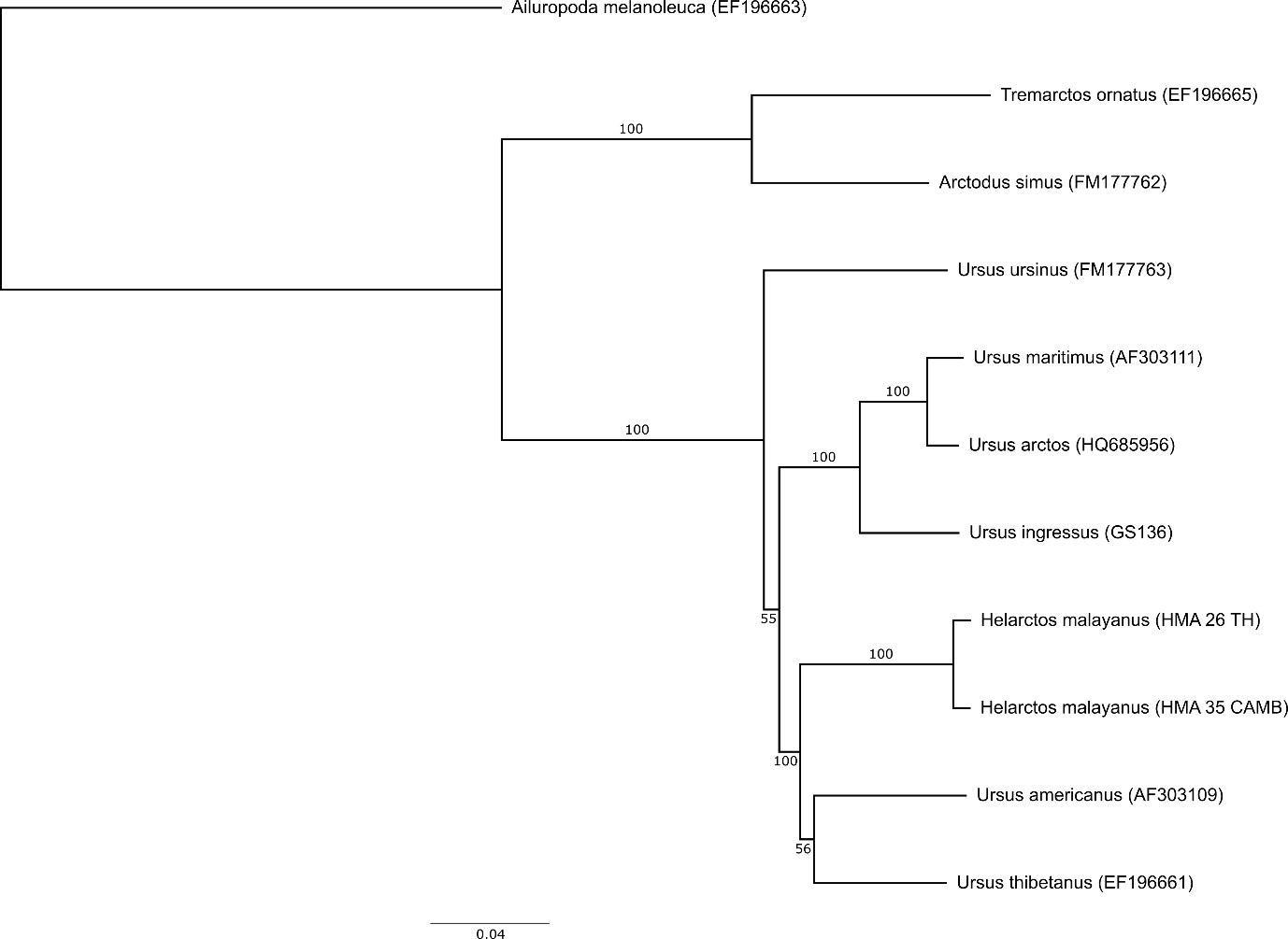


**Suppl. Figure S3**. Maximum-likelihood tree based on sun bear mitochondrial sequences


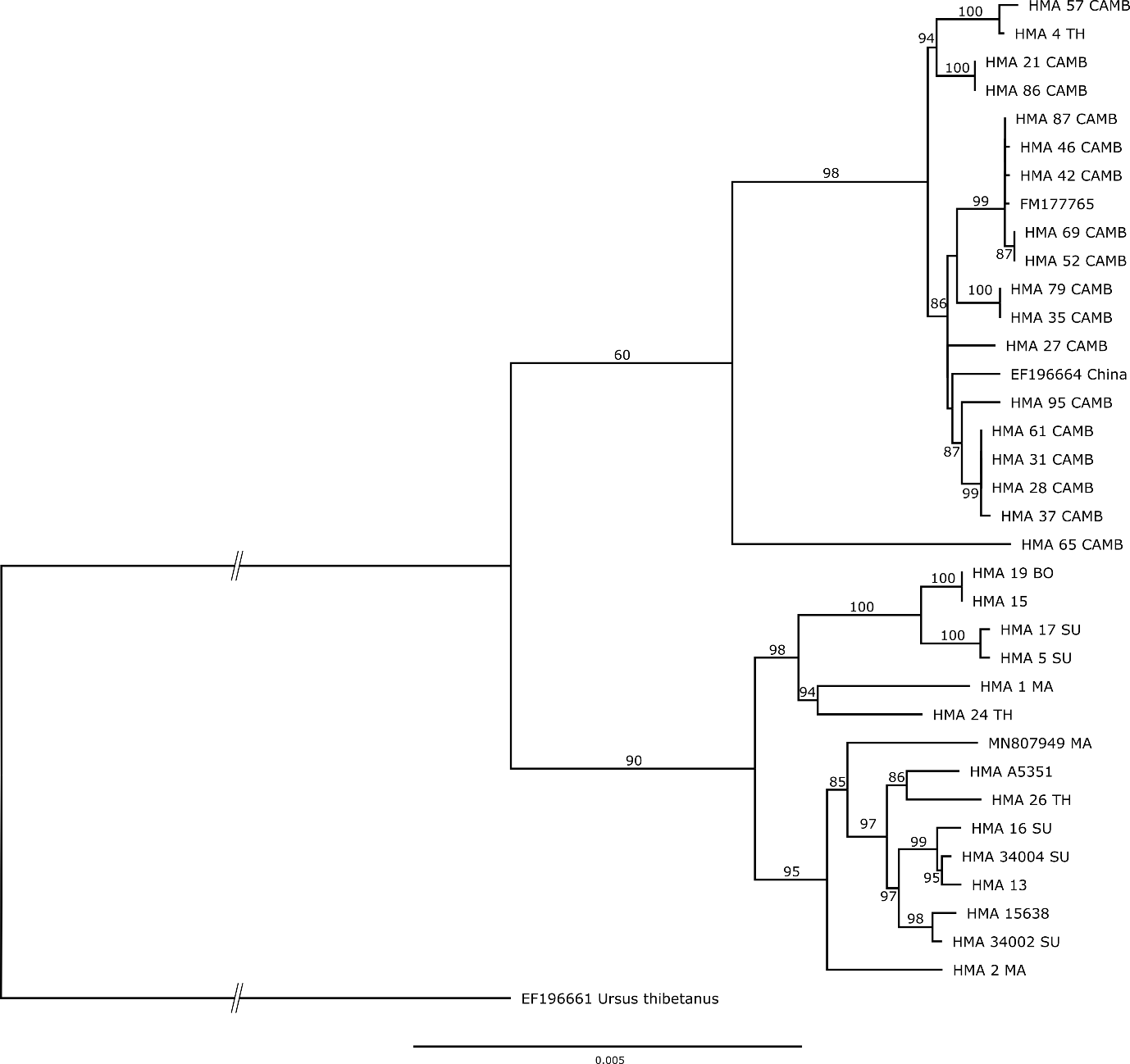

Supplement: Supplementary file 1 — Appendix S1 [file ECE3-13-e9969-s001.docx]
